# Uncertainty and precaution in hunting wolves twice in a year: Reanalysis of Treves and Louchouarn

**DOI:** 10.1371/journal.pone.0301487

**Published:** 2024-06-12

**Authors:** Glenn E. Stauffer, Erik R. Olson, Jerrold L. Belant, Jennifer L. Stenglein, Jennifer L. Price Tack, Timothy R. van Deelen, David M. MacFarland, Nathan M. Roberts

**Affiliations:** 1 Office of Applied Sciences, Wisconsin Department of Natural Resources, Rhinelander, WI, United States of America; 2 Department of Forest and Wildlife Ecology, Northland College, Ashland, WI, United States of America; 3 Department of Fisheries and Wildlife, Michigan State University, East Lansing, MI, United States of America; 4 Department of Forest and Wildlife Ecology, University of Wisconsin, Madison, WI, United States of America; 5 Department of Conservation and Wildlife Management, College of the Ozarks, Point Lookout, MO, United States of America; University of Ferrara Department of Life Sciences and Biotechnology: Universita degli Studi di Ferrara Dipartimento di Scienze della Vita e Biotecnologie, ITALY

## Abstract

Management of wolves is controversial in many jurisdictions where wolves live, which underscores the importance of rigor, transparency, and reproducibility when evaluating outcomes of management actions. Treves and Louchouarn 2022 (hereafter TL) predicted outcomes for various fall 2021 hunting scenarios following Wisconsin’s judicially mandated hunting and trapping season in spring 2021, and concluded that even a zero harvest scenario could result in the wolf population declining below the population goal of 350 wolves specified in the 1999 Wisconsin wolf management plan. TL further concluded that with a fall harvest of > 16 wolves there was a “better than average possibility” that the wolf population size would decline below that 350-wolf threshold. We show that these conclusions are incorrect and that they resulted from mathematical errors and selected parameterizations that were consistently biased in the direction that maximized mortality and minimized reproduction (i.e., positively biased adult mortality, negatively biased pup survival, further halving pup survival to November, negatively biased number of breeding packs, and counting harvested wolves twice among the dead). These errors systematically exaggerated declines in predicted population size and resulted in erroneous conclusions that were not based on the best available or unbiased science. Corrected mathematical calculations and more rigorous parameterization resulted in predicted outcomes for the zero harvest scenario that more closely coincided with the empirical population estimates in 2022 following a judicially prevented fall hunt in 2021. Only in scenarios with simulated harvest of 300 or more wolves did probability of crossing the 350-wolf population threshold exceed zero. TL suggested that proponents of some policy positions bear a greater burden of proof than proponents of other positions to show that “their estimates are accurate, precise, and reproducible”. In their analysis, TL failed to meet this standard that they demanded of others.

## 1 Introduction

Management of wolves is controversial in many jurisdictions where wolves live, and that controversy sometimes results in back-and-forth judicial rulings and abrupt shifts or reversals in proposed or realized management actions [1]. In Wisconsin, USA, protected status of wolves under the Endangered Species Act (ESA) has changed multiple times in the past two decades. In November 2020, the U.S. Fish & Wildlife Service published a rule which removed the gray wolf (*Canis lupus*)from the federal list of endangered species across the lower 48 states, including Wisconsin, with an effective date of January 4, 2021. Because Wisconsin law requires a hunting and trapping season when wolves are not protected under federal law (WI Stat § 29.185), the Wisconsin Department of Natural Resources (DNR) announced ahead of this delisting the intent to hold a fall 2021 wolf harvest season. However, the Wisconsin statute mandating a harvest season sets that season to open on the first Saturday of November and extend to February 28, or until the harvest quota is achieved. During the only three previous wolf harvest seasons (2012–2014), the harvest quota was always achieved before 25 December such that harvest seasons did not extend into February. In early February 2021, after the January 2021 delisting, the Wisconsin Institute for Law and Liberty (WILL) filed a lawsuit on behalf of Hunter Nation, a hunting rights advocacy group, to compel the DNR to hold a wolf harvest season immediately, before the February end of the statutory harvest season. The court sided with Hunter Nation and consequently, Wisconsin expedited the opening of a judicially mandated wolf harvest season in February 2021, well outside the date range of previous harvest seasons in Wisconsin.

The February 2021 harvest quota was set at 200 wolves, 81 of which were allocated to the six Ojibwa tribes of Wisconsin per their treaty rights in Wisconsin’s ceded territories. The Ojibwa tribes elected to harvest no wolves. The season lasted only three days, including a mandatory 24-hour closure notice, but by season’s end the total harvest was 218 wolves, about 83% over the state’s portion of the overall quota (119), or 9% over the overall quota. The greater-than-expected harvest, occurring well outside the time-frame of the traditional hunting season, generated concern about further impacts of a subsequent legally-mandated harvest season planned for fall 2021. The harvest quota for the proposed fall 2021 season was set at 130 wolves.

Treves and Louchouarn [2] (hereafter TL) urged precaution in harvesting wolves twice in a calendar year, and suggested that a zero death toll would be precautionary. TL used a simple one-step population model to predict wolf population size in Wisconsin, given a range of harvest scenarios, including a zero harvest scenario, for a hypothetical fall 2021 hunting season. They used 3 thresholds for evaluating potential wolf population decline in response to fall 2021 hunting and trapping mortality: (1) the 1999 Wisconsin wolf management plan population goal of 350 wolves; (2) the 250-wolf threshold for statutory listing under the state threatened and endangered species act; and (3) state extirpation, which they defined as <2 wolves. TL concluded that even a zero harvest scenario could result in the wolf population declining below the 1999 population goal of 350 wolves and that a fall harvest of >16 wolves was expected to result in a “better than average possibility” that the wolf population size would decline below that same threshold. TL further concluded there was a >50% chance of crossing the second threshold of 250 with a harvest as small as 113 wolves, and a >50% chance of state extirpation by April 2022 with a harvest as low as 359 wolves.

The planned fall 2021 hunt was judicially blocked just prior to the planned opening date, resulting in a realized zero harvest quota. The winter 2021–2022 population estimate therefore provides a direct means to evaluate the explicit quantitative prediction from TL for a zero harvest scenario. Here, we provide a critique of TL and show that the risk of population decline concluded by TL was exaggerated as a consequence of unsubstantiated assumptions and mathematical and other methodological errors in specifying and calculating model parameters. In particular, TL 1) exaggerated non-hunting mortality; 2) excessively reduced the possible proportion of packs that produced pups; 3) wrongly halved the number of pups that survived to November; and 4) subjected individuals potentially killed in a fall hunt to an additional 5 months of mortality. In every case, these errors are in the direction of exaggerating declines in wolf population size, and cumulatively they strongly influenced calculation of risk in TL and resulted in incorrect and potentially consequential conclusions about wolf population response. That these faulty conclusions already have been offered as scientific evidence in state litigation involving wolf management in Wisconsin underscores the importance of rigor, transparency, and reproducibility when evaluating outcomes of proposed or realized management actions.

## 2 Methods and materials

TL used a one-step projection model to predict ending population size, given a range of harvest scenarios from 0 to 600 wolves. The model specified in Eq 1 of TL is *N*_*t*+1_ = *N*_*t*_ + *R*_*t*_ − *M*_*t*_ − *H*, where *N*_*t*_ is population size on 15 April 2021, *N*_*t*+ 1_ is predicted population size on 14 April 2022, *R*_*t*_ is the number of pups that survived to November (the time of the proposed harvest), *M*_*t*_ is the number of dead wolves in year *t* (although not explicitly stated, it is actually the number of wolves that die from causes other than hunting), and *H* is the number of wolves harvested in year *t*. *R*_*t*_ is defined as *B*_*t*_ × *L* × *S*, where *B*_*t*_ is the number of breeding packs, *L* is litter size, and *S* is pup survival probability from birth to November. As explained later, although the text of TL defines *R*_*t*_ = *B*_*t*_ × *L* × *S*, in their Table S2, TL actually defined *R*_*t*_ as *B*_*t*_ × *PPN* × *L* × *S*, where *PPN* is the proportion of packs that produce pups.

We assessed assumptions and decisions that TL made to specify parameters for this model, and where we found parameter specification indefensible or biased, we specified more defensible parameter values. Table 1 compares the parameterization of TL, the corrected parameterization used in our re-analysis, and the rationale for our chosen values. We then used the projection model from TL to predict ending population size for both scenarios. As in TL, in our specification of *N*_*t*_ we subtracted the presumed 42 wolves living largely on tribal reservations. We do not suggest that this forecasting methodology is optimal or the best approach for predicting wolf population size in response to hunting mortality, but we retained this framework to make our predictions directly comparable to those from TL. We compared results from both scenarios (TL parameterization and our alternative analysis with corrected parameter values) graphically and by calculating the probability of crossing each of the defined thresholds, for several harvest scenarios. For display purposes we show 3600 simulation samples, consistent with TL, but for inference about probability of crossing thresholds, we simulated 1 million samples. R code for performing the simulations is available in S1 File.

**Table 1:** Comparison of model parameterization from Treves and Louchouarn [2] with values used in this paper for reanalysis. In parameter notation, dU represents a discrete uniform distribution (integer values between specified bounds), N represents a normal distribution, Be represents a beta distribution, and Bern represents a Bernoulli distribution.

Parameter	Description	TL	This paper	Rationale
*N*_*t*_	Initial N (min count)	*U*(695,751)	Same	For illustration, we used the values from TL, although these values do not represent empirical counts.
*N*_*t*_	Initial N (occupancy model)	1075 + (*dU*(−496, 756) + *dU*(−496, 756))/2 − 218	*N*(1126, 110)	TL specification is skewed toward extreme values—our specification closely approximates the empirical 2021 population estimate.
*D*	Annual mortality rate	*dU*(0.38,0.56)	*N*(0.25,0.019)	Stenglein et al. (2015, 2018) provided more plausible and defensible mortality estimates.
*B*_*t*_	Packs in population	*dU*(74,167)	245 − 51 = 194	TL twice accounted for non-breeding packs, and *B*_*t*_ = 74 represents improper and misleading extrapolation from a very small sample.
*PPN*	Proportion of packs reproducing	{(*dU*(1, 17) + *dU*(1, 17)) − 2}/100 + 0.52	*Be*(34.51, 16.24)	Standard statistical distribution (mean = 0.68) to approximate TL
*L*	Litter size	{*TRUNC*(*dU*(3, 6)+ *dU*(3, 6)}/2+ 0.3)^a^	*Be*(3, 2) × (6 − 3) + 3	Standard statistical distribution (mean = 4.8) to approximate [3]
*S*	Pup survival to Nov	{(*dU*(0, 16) + *dU*(0, 16) + *Bern*(0.25) × *dU*(3, 40)}/100	*Be*(24, 25.6)	Standard statistical distribution (mean = 0.484) that approximates the histogram of annual survival, scaled to 7 months, from Table 6.3 of [4].
*R*_*t*_	Total pups surviving to Nov	*B*_*t*_ × *PPN* × *L* × *S*	Same	We accept the equation from TL.
*H*	Harvest	*dU*(0, 300) + *dU*(0, 300)	Same	We used the harvest scenarios from TL.
*M*_*t*_	Non-harvest deaths	*D* × (*N*_*t*_ + *R*_*t*_/2)	*M*_*t*1_ + *M*_*t*2_^b^	TL improperly counted some deaths twice.
*N*_*t*+1_	Ending population	*N*_*t*_ + *R*_*t*_/2 − *M*_*t*_ − *H* − *tribal*	*N*_*t*_ + *R*_*t*_ − *M*_*t*_ − *H* − *tribal*	TL improperly doubled pup mortality (i.e., *R*_*t*_/2).

^a^ Note that the addition of 0.3 here does not affect the truncated value.

^b^ *M*_*t*1_ + *M*_*t*2_ = *N*_*t*_ × *D* + [1 − (1 − *D*)^5/12^] × (*R*_*t*_ − *H*)

Several clarifications about parameterizations specified in TL are needed. First, the parameterization in Table 1 reflects parameterization specified in Table S2 of TL, which as far as we could determine specified the actual analysis, but which sometimes differed slightly from parameterization specified in the text of TL. For example, the text of TL specified, somewhat vaguely, *PPN* = (0.55–0.89, *mean* = 0.72), but Table S2 of TL specifies (((*RANDBETWEEN*(1,17) + *RANDBETWEEN*(1,17)) − 2)/100) + 0.52, which has mean 0.68 and is truncated at [0.52,0.84]; note that *RANDBETWEEN* is equivalent to a discrete uniform distribution. Second, for pup survival (*S*), TL specified a mixture of several distributions resulting in a highly asymmetric distribution with a mean of 0.2, and a range of 0.05–0.72, citing Thiel et al. [3]. Although the mean is 0.2, the most likely value is 0.17, and the 97.5th percentile is 0.49. Moreover, the estimate is incorrect, and TL posted a comment on their paper (7 Aug 2022) acknowledging the error, stating therein that their conclusions nonetheless remain unchanged [2]. In their correction, TL cited *S* = 0.29 (range = [0.14–0.58]) as mean (min/max) from Table 6.3 in Wydeven et al. [4], but they do not define this distribution analytically. Consequently, and because TL stated that their conclusions did not change, we used *S* as initially specified in TL for our replication of the TL analysis (but in our discussion we point out further problems with this choice for *S*). Third, TL claimed to compare predictions for two scenarios, one where *N*_*t*_ represented the traditional census method, and one where *N*_*t*_ represented the population estimate from the newer scaled occupancy model [5]). However, what TL presented as a count from the “traditional census method” for 2021 is not an empirical count produced by the Wisconsin DNR (no such count exists) but rather a predicted count that assumes certain hypothetical population growth rates [6]. Without endorsing the predicted count of *N*_*t*_ as correct, we nonetheless specified the same value for our current analysis to make our projections directly comparable to those of TL. Fourth, the distribution presented by TL as representing the new census method (the scaled occupancy approach from [5]) places too much probability mass in the distribution tails, and thus exaggerates uncertainty. In our reanalysis, we specified a distribution that closely matches the empirical scaled occupancy estimate from [7] for *N*_*t*_ (Table 1).

## 3 Results and discussion

With parameterizations reflecting as closely as possible those in TL, we were able to closely reproduce the prediction results of TL. For example, with harvests of 0, 130, and 300, TL predicted mean population sizes of 361, 231, and 66, respectively, for the “over-winter counts”, with *SD* = 44–45 in all cases; similarly, our replication of TL predicted population sizes of 360, 230, and 60, respectively, with *SD* = 45 in each case.

**Fig 1:**
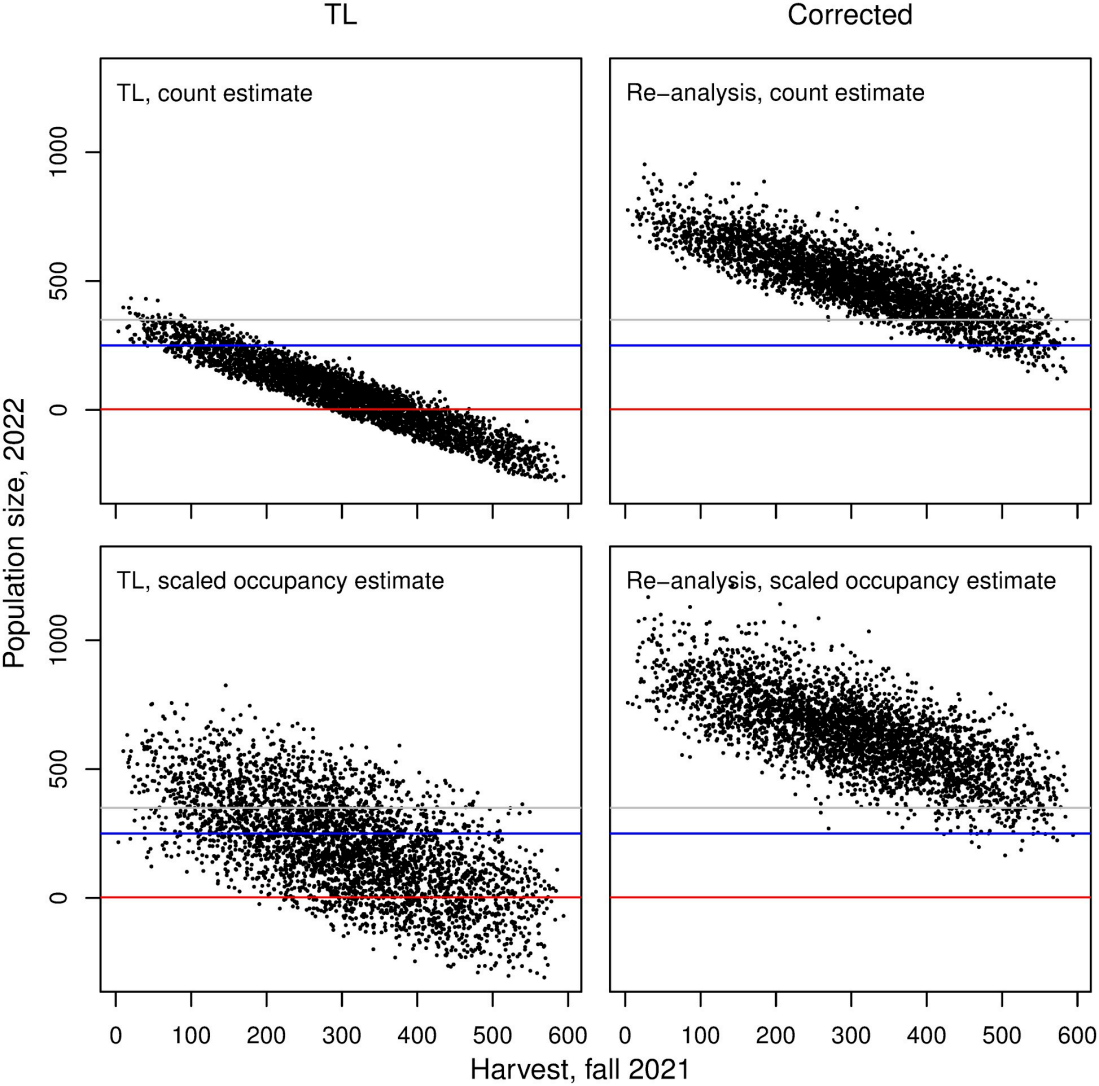
Simulation outcomes for wolf population size. Simulation results predicting wolf population size in April 2022, assuming an uncertain fall harvest of 0–600. The three horizontal lines represent population thresholds of 350 (1999 population goal), 250 (relisting threshold), and 2 (extirpation) considered in Treves and Louchouarn [2].

With corrected parameter values, mean predicted final population sizes were substantially greater than in TL, and probability of extirpation was zero for all harvest scenarios (Fig 1, Table 2). Only in scenarios with simulated harvest of 300 or more wolves did probability of crossing even the first population threshold of 350 exceed zero (Table 2).

**Table 2:** Probability of crossing 3 population thresholds defined in Treves and Louchouarn (TL) [2] for various fall 2021 harvest scenarios when starting population is specified as a predicted “count” (Count) or a scaled occupancy estimate (Occ; Stauffer et al. [5]), and using parameterization from TL or corrected parameterization from this paper (Current).

Harvest	Threshold^a^	TL—Count^b^	TL—Occ	Current—Count	Current—Occ
Harvest = 0					
	Management	0.432	0.166	0.000	0.000
	Relisting	0.000	0.038	0.000	0.000
	Extirpation	0.000	0.000	0.000	0.000
Harvest = 130					
	Management	0.998	0.461	0.000	0.000
	Relisting	0.641	0.222	0.000	0.000
	Extirpation	0.000	0.000	0.000	0.000
Harvest = 300					
	Management	1.000	0.836	0.001	0.002
	Relisting	1.000	0.638	0.000	0.000
	Extirpation	0.108	0.092	0.000	0.000
Harvest = 500					
	Management	1.000	0.990	0.652	0.125
	Relisting	1.000	0.946	0.092	0.015
	Extirpation	1.000	0.518	0.000	0.000
Harvest = 600					
	Management	1.000	1.000	0.957	0.395
	Relisting	1.000	0.990	0.585	0.103
	Extirpation	1.000	0.752	0.000	0.000

^a^Thresholds were: Management, 350; Relisting, 250; Extirpation, <2

^b^Although TL refer to this count as the “traditional minimum count”, it is not an empirical count but rather a projection from [6], assuming certain hypothetical population growth rates.

TL made quantitative predictions about ending population size in spring 2022, assuming zero harvest. The actual estimated spring 2022 population size, after realized zero harvest in fall 2021, was 972 (95% credible interval = 812–1,193) [8]. Using the parameter values from TL, and assuming the TL specification for the initial population size, as estimated by the occupancy model, the predicted spring 2022 population size was 500 (95% credible interval = 218–784), or approximately 51.5% of the estimated spring 2022 population. When TL recalculated projections based on their corrected pup mortality as described in the comment on TL [2], the predicted population under a zero-harvest scenario was 648 (range 291–1017), or about 67% of the estimated spring 2022 population, and about 2% of predicted values crossed below the threshold of 350 wolves (see 7 Aug 2022 online comment on [2]). Although TL stated in their comment that their conclusions did not change after the correction, we note that this probability was substantially lower than before the correction (Table 2). Using our corrected parameter specifications and calculations (Table 1), we predicted a final population size of 911 (95% credible interval = 708–1,113), or approximately 93.7% of the empirically estimated spring 2022 population. We are aware that the an author of TL has asserted that the spring 2022 wolf population estimate represents an over-estimate [9]. Though beyond the scope of this paper to address errors and misrepresentations in [9], we encourage readers to discern assertion from facts or science and recognize that an assertion does not negate the errors we have identified in TL and describe in following sections. The errors detailed in the following sections primarily represent fundamental errors in mathematics regardless of subsequent population estimates or flawed conclusions in [9].

### 3.1 Specifying mortality probability

TL used an *ad hoc* method to approximate a range of annual mortality rates (*D*) for a year without Endangered Species Act protections and without a wolf hunt. Assuming a wolf year from 15 Apr in year *t* to 14 Apr in year *t+1*, they calculated *D* = *M*_*t*_/(*N*_*t*_+0.5×*R*_*t*_), where parameters are as described above.

The estimates for *D* provided in Table 1 of TL are problematic for several reasons. First, the equation to calculate *D* provided in the caption to TL Table 1 is (*N*_2021_ − *N*_2020_)/(*N*_2020_ + 0.5 × *R*_2020_), which cannot be correct because it yields negative *D*, because *N*_2021_ < *N*_2020_ (e.g., 969–1034 = −65). We suspect this reflects a typographical error and TL meant to say (*N*_2020_ − *N*_2021_)/(*N*_2020_ + 0.5 × *R*_2020_), although this formulation would then produce negative *D* if *N*_*t*+1_ > *N*_*t*_. Additionally, the assumption in Table 1 of TL that *M*_*t*_ = *N*_*t*_ − *N*_*t*+1_ is self-evidently problematic, and differs with how TL previously defined *M*_*t*_. For our current analysis, rather than relying on an *ad hoc* estimate for *D*, we used an estimate derived from rigorous peer-reviewed analyses of Wisconsin wolves [10, 11]. Below, we provide further discussion and defense of these peer-reviewed analyses.

Second, the min/max estimates presented by TL for *N*_2021_ were implied to be estimates equivalent to the previous year’s count estimate produced by the Wisconsin DNR, but the estimates are not comparable. The Wisconsin DNR did not generate a count in 2021, and TL would not have had access to the data required to generate such an estimate. Instead, the min/max estimates presented by TL for *N*_2021_ appear to derive from deterministic calculations assuming certain hypothetical values for annual population growth [6]. The range 695–751 does not represent a 95% confidence interval but rather extremes representing the bounding values for population growth assumed by Treves et al. [6]. We were unable to reproduce the estimates for *D* in TL Table 1, even using the values specified by TL. It is unclear what values TL actually used to calculate *D*.

Furthermore, in an attempt to justify their specification for *D*, TL stated that there is little scientific consensus on annual mortality rates among Wisconsin wolves, but then they dismissed or ignored several rigorous, defensible, peer-reviewed, and remarkably similar estimates from Wisconsin and neighboring states in the upper Great Lakes region [4, 10–13]. In contrast, TL uncritically accepted implausible conclusions that a wolf population could sustain an annual population growth rate of 1.13 while suffering an annual mortality rate of 0.46 [14, 15]. In particular, TL dismissed the annual mortality estimate of 0.241 (*SD* = 0.019) provided by Stenglein et al. [10], because it “seems low” and because “…that study failed to account for several confounding variables and took unjustified steps in analyses” [2, p. 10]. TL then specifically criticized: 1) a variable for change of slope in the year 2004; 2) unaccounted changes in census methodology; 3) pooling non-human causes of death with unknown causes of death; 4) failure to acknowledge potential differences in mortality for collared and uncollared wolves; 5) failure to acknowledge evidence that wolf survival and population growth declined when ESA protections were lifted; and 6) failure to account for seasonal changes in incidence of wolf mortality. They concluded that the estimate from Stenglein et al. [10] was “certainly too low given the conditions between 3 November 2020 and 13 April 2021.” It is unclear whether the conditions to which they refer relate to their criticism 6 or to the Feb 2021 wolf hunt. Mortality from the wolf hunt is already accounted for in the starting population for the population projection and thus should not be considered for non-hunting annual mortality.

The claimed issues in criticisms 1–3 are not relevant to the annual survival estimate from Stenglein et al. [10], nor are they germane to either TL or this response. Criticism 4 raises an important question, but the assertion is false; Stenglein et al. [10] did address this issue explicitly by citing Stenglein et al. [11], who rigorously evaluated the extent to which mortality estimated from radio-collared wolves might underestimate population level mortality, given population growth and recruitment rates. Stenglein et al. [10] concluded that only minor adjustment was needed (i.e., annual mortality was 25% instead of 24%). Moreover, the differences in mortality rates suggested by Treves et al. [14] are implausible given the estimated annual population growth rates, estimated empirically from extensive snow-tracking data [4]. Criticism 5 relates to the claims made in Chapron and Treves [16], which have been thoroughly discredited by multiple authors [17–19], and responses by Chapron and Treves [20, 21] to those criticisms did not refute or adequately address them. Moreover, Stenglein et al. [10] used a temporal spline for year that captured potential interannual differences in mortality resulting from changes in legal protections. Criticism 6 is also false; Stenglein et al. [10] fitted a temporal model that allowed mortality hazard to vary weekly throughout the year. TL failed to demonstrate here or in any of their citations that the annual mortality estimate provided by Stenglein et al. [10] is implausible or unreliable, and we conclude that estimates for wolves in Wisconsin reported by Stenglein et al. [10], incorporating Stenglein et al. [11], are the most plausible and defensible estimates available. In contrast, the estimates of *D* presented in TL exaggerate annual mortality by as much as 100%.

As noted above, TL recognized that pup survival (*S*) was misspecified in their paper, and posted an online comment on the the journal website (7 Aug 2022) specifying a somewhat ambiguously defined correction of *S* = 0.29 (range = 0.14–0.58) from Table 6.3 in Wydeven et al. [4]. Regardless of it’s precise definition, this value for *S* is still problematic for at least two reasons. First, if the new distribution with mean 0.29 has the same shape as the old distribution, then probability mass is concentrated in the lower end of the range with mode < mean. Secondly, and more importantly, the *ad hoc* estimates in Table 6 of Wydeven et al. [4] represent survival estimates from birth to the end of a pup’s first winter, a period of approximately 12 months. TL acknowledge that their model requires estimates for pup survival until November, which, assuming pups are born in April, is a period of about 7 months. To use these ad hoc estimates, TL should have converted them to 7-month estimates ($S_{7mo}=S_{12mo}^{\left(7/12\right)}$) which would result in a mean of 0.484 with [*min*, *max*] = [0.318, 0.728]. We used a beta distribution with mean 0.484 to approximate *S* (Table 1), which appropriately accounts for 7 months of survival but is somewhat more conservative than Table 6 in [4] in the right tail of the distribution. Alternatively, TL could have used the estimate of 0.72 (95% C.I. = 0.51–0.94) from 84 radio-collared wolves [4]. Despite the correction, *S* in TL remains strongly and negatively biased.

### 3.2 Proportion of packs reproducing

In the calculations for the *ad hoc* estimate of *D* in Table 1 of TL, *B*_*t*_ = 245 represents all 256 presumed packs in the population, minus the 11 packs inhabiting tribal reservations (refugia from hunting), and the fact that not all packs reproduce each year is captured in the parameter *PPN* = 0.72 [range = 0.55–0.89]. But, TL *twice* used *PPN* to reduce the number of packs that produced pups. TL multiplied 245 by the upper bound of *PPN* (245 × 0.89 = 218), and then subtracted 51 presumed breeding females (we explain below why 51 might represent a positive bias) to derive an upper bound of 167 for *B*_*t*_. However, in their parameter specifications in Table S2 of their paper, TL then derived *R*_*t*_ = *B*_*t*_ × *PPN* × *L* × *S*. To put it succintly, TL calculated *B*_*t*_ = *N*_*packs*_ × *PPN*_*max*_ − 51, and then calculated *R*_*t*_ = (*N*_*packs*_ × *PPN*_*max*_ − 51) × *PPN* × *L* × *S*. We also note that the distribution specified for *PPN* in Table S2 of TL produces a mean of 0.68 rather than 0.72 stated in TL.

The deduction of 51 packs by TL was based on the assumption that 23% of the harvested wolves were pregnant females, rather than from an examination of the complete harvest data. TL cited a small, non-random sample of 22 wolves, and noted that 65% of adult females and 50% of yearling females were pregnant, but they did not state exactly what proportion of the sample was females. Although not explicitly stated, it was misleadingly implied that 50–65% of the sample was pregnant females, and that by comparison, 23% was a conservative estimate. In fact, TL used this implied conclusion that 65% of the *harvest* (not 65% of the *females in the harvest*) were pregnant to assert a lower bound of only 74 packs that reproduced. In their simulations, they then assumed *B*_*t*_ ∼ *Uniform*(74, 167), and they noted that *B*_*t*_ = 12 and *B*_*t*_ = *N*_*packs*_ × 0.89 = 218 were “*implausible extreme values*”, which they did not use in their simulations. The use of the uniform distribution was justified by TL in their caption to their Fig 2 (“…the uniform, uninformative distribution allows the data to influence the result rather than our preconceived notions of what is typical in biological distributions”). This statement is incorrect and misleadingly implies that the specified uniform distribution was an uninformative prior for *B*_*t*_, and that fitting data to a statistical model likelihood resulted in an updated and more informative posterior estimate for *B*_*t*_. TL did not fit data to a model, and consequently, it is meaningless to speak of uninformative prior distributions, as if there were a Bayesian analysis that could in any way provide updated posterior parameter estimates. Rather, the projection in TL was a simple simulation where the specified distributions for each of the parameters were arithmetically combined to produce a result that depended directly on the specified inputs. In other words, the specified parameter inputs *were themselves the data* and those specified parameters *directly* and *deterministically* influenced the results. In the case of *B*_*t*_, the specified uniform distribution was premised on consequential assumptions and included a calculation error, both of which absolutely influenced the results in a specific direction (fewer breeding packs).

### 3.3 Pup production to November

In Equation 1, TL presented their simple one-step model of population size change as *N*_*t*+1_ = *N*_*t*_ + *R*_*t*_ − *M*_*t*_ − *H*. In Table S2 of TL, they presented a form of the model presumably used in the simulations. That model was *N*_*t*+ 1_ = (*N*_*t*_ + *B*_*t*_ × *PPN* × *L* × *S* × 0.5) × (1 − *D*) − *H*. Allowing *R*_*t*_ = *B*_*t*_ × *PPN* × *L* × *S* (note: *R*_*t*_ was not defined this way in Equation 2 of TL, but was so defined in Table S2 of TL), and allowing *M*_*t*_ = *D* × (*N*_*t*_ + *R*_*t*_/2) as in equation 3 of TL, and then rearranging terms, yields a one-step model *N*_*t* = 1_ = *N*_*t*_ + *R*_*t*_/2 − *M*_*t*_ − *H* where the number of pups surviving until November is only half that stated in Equation 1. To reiterate, the projection model used by TL inappropriately halved pup production compared to the model specified in the text of TL; instead of using *R*_*t*_ as specified in Equation 1 of TL, TL used *R*_*t*_/2.

### 3.4 Subjecting harvested individuals to additional mortality

TL defined *M*_*t*_ = *D* × (*N*_*t*_ + *R*/2) as the number of wolves that die from *t* to *t* + 1 (excluding harvest), but this definition exaggerates mortality if there is a fall harvest, because it does not subtract out harvested wolves from *N*_*t*_. TL recognized this positive bias [2, p. 11], but dismissed it by suggesting that any bias was offset by also dismissing unreported deaths and excess legal killing. There may be unreported deaths and illegal killing, but TL did not provide convincing evidence that the magnitude was comparable to the positive bias introduced by counting mortalities twice, or that such unreported deaths generate substantial negative bias in mortality estimates. Moreover, TL already presumably accounted for the possibility of additional deaths in the consideration of *H*. TL implied that unreported deaths necessarily result in underestimates of *D*, which is not typically the case with analyses that rigorously account for unobserved mortality [10].

Assuming for simplicity that there are 7 months of non-harvest mortality prior to a fall hunt, a fall hunt where harvest is accomplished relatively quickly (this is usually the case—in three previous hunts in WI in 2012–2014, 75% of the total harvest was achieved in 5 weeks, 3 weeks, and 2 weeks, respectively [22]), and then 5 more months of mortality to the end of the time-step, a better accounting of non-hunt mortality is as follows. Let *M*_*t*1_ = [1 − (1 − *D*)^7/12^] × *N*_*t*_ be the number of individuals that die prior to the hunt and *H* be the number of individuals that are harvested. The number that die after the harvest is then *M*_*t*2_ = [1 − (1 − *D*)^5/12^] × (*N*_*t*_ − *M*_*t*1_ + *R*_*t*_ − *H*). Combining expressions and collecting terms results in *M*_*t*_ = *M*_*t*1_ + *M*_*t*2_ = *N*_*t*_ × *D* + [1 − (1 − *D*)^5/12^]×(*R*_*t*_ − *H*). Obviously, *M*_*t*_ decreases as *H* increases, so the bias that results from failing to subtract out *H* is greatest when *H* is large. This formulation assumes completely additive harvest mortality, which is unlikely, as some degree of compensation is both theoretically expected and empirically estimated [10, 23–25]. In contrast, the formulation of TL allowed some deaths to be counted twice, if there is a harvest (which essentially imposed artificial super-additive mortality).

### 3.5 Other errors or misstatements

TL stated that Wisconsin’s new occupancy approach to estimated wolf population size was unpublished and not peer-reviewed. This is false because the general methodology was published before the submission of TL [5].

TL also stated that they “present the first estimates for annual mortality rate between 15 April 2020 and 14 April 2021.” The *Uniform*(0.38, 0.56) distribution that they presented is not a rigorous mortality estimate, but rather an *ad hoc* back-of-the-envelope calculation that we were unable to replicate, and that differs substantially from numerous other published and more rigorous estimates [4, 10–13].

TL stated that “The traditional census method had a reliable slope as judged by its r-squared value, twice as reliable as the new census method”. First, this is a mischaracterization of statistical reliability in the face of uncertainty. In a simulation exercise as in TL, one could make the correlation perfect by ignoring uncertainty entirely, but that does not make the slope more reliable. Second, as discussed previously, the population size estimates presented by TL do not represent the traditional census method but rather deterministically calculated bounds assuming certain hypothetical values for annual population growth [6].

The introduction and conclusion of TL stated that they used “Bayesian concepts” to account for uncertainties, but it is unclear what TL meant by that other than simply using Bayesian terminology [2, p. 4]. TL did not estimate parameters by fitting data to a model likelihood, and they provided no information or data that could update model parameters at all. Rather, TL conducted a simple simulation where uncertain parameters were used to calculate an uncertain but deterministic metric of interest. There is perhaps nothing inherently wrong with such an approach, but there also is nothing Bayesian about it.

TL engaged in a lengthy discussion about why they were justified in deducting annual mortality before the wolf hunt, and they stated that doing so treated wolf harvest as purely additive. First, it exceeded purely additive mortality, as explained above, because the calculation of deaths included a math error that allowed harvested wolves to be counted twice. Second, even the assumption of complete additivity is speculative—from a theoretical and empirical standpoint it is likely that harvest mortality is partially compensatory [10, 23–25].

In their conclusion, TL wrongly cited Treves et al. [26] as the source for the annual mortality estimate of 0.235 from Stenglein et al. [13] (also, the provided DOI for this citation was incorrect), and then went on to say that this estimate “is only plausible for 2020 if one accepts a drastic rise in population size from 2020 to 2021” [2, p. 14]. TL did not justify this assertion. In fact, an annual mortality estimate of 0.235 seems entirely reasonable in light of other published survival estimates from Wisconsin and Michigan [4, 12].

TL cited Artelle et al. [27] suggesting that many wildlife hunting plans lack clear objectives, independent review, and transparency, and then expressed regret that the Wisconsin DNR was favorably scored in [27], citing several controversial papers [6, 28] as evidence that the state of Wisconsin did not merit such a favorable review. TL further accused Wisconsin of inflating wolf quotas and “distorting sound science-informed management”, and they insinuated that the Wisconsin DNR (and other agencies) “pick and choose the evidence they wish to use based on their personal or organizational values” [2, p. 15]. This is a remarkable insinuation, given the parameter specification in TL that resulted in greatly exaggerated risk of wolf population decline in response to a wolf hunt. Although the authors may not have intended it, math errors and selected parameterizations in TL were consistently biased in the direction that maximized mortality and minimized reproduction (adult mortality, pup survival, halving pup survival to November, twice discounting the number of breeding packs, and counting harvested wolves twice among the dead). The result was erroneous conclusions that were not based on the best available or unbiased science.

## 4 Conclusions

We appreciate that TL have expressed concern about the risks of wolf population decline in the face of an unprecedented February hunt followed by a fall hunt in the same calendar year, and we agree that such risks should be carefully evaluated and weighed. However, we have demonstrated serious flaws in the analysis of TL that invalidate their conclusions about the severity of those risks. Based on their flawed analysis, TL asserted that “…a zero death toll would be precautionary. Proponents for high quotas bear the burden of proof that their estimates are accurate, precise, and reproducible” [2, p. 1], and that “Anyone who steps away from the precautionary approach must present stronger evidence for their more optimistic view” [2, p. 17]. It is notable that TL claimed that a higher burden of proof applies to science to support some policy positions but not other policy positions. Effective resource management relies upon accurate, unbiased scientific information with rigorous estimates of uncertainty, coupled with evaluation of multiple, often conflicting, societal objectives. Faulty or biased science, regardless of the directionality of the error, is harmful to policy decisions, and policy recommendations based on such science cannot be considered “precautionary”. Furthermore, “precautionary” itself implies a value judgment that is defined by policy, not science. The role of science is to provide robust, reliable information, not to define the directionality or desired level of precaution. All scientists bear an equal responsibility to ensure, in the words of TL, “…that their estimates are accurate, precise and reproducible” [2, p. 1]. Failure to meet these basic scientific standards erodes public confidence in science and the decisions it is intended to support. In this case, TL have failed to meet the standards that they demanded of others.

Apart from the serious flaws we documented in TL, we make two related and important points. First, as pointed out by one of our reviewers, we believe that our work exposes a serious failure in the peer-review process. Ostensibly, the review process is meant to ensure that published scientific papers are scientifically sound. In this case, reviewers and editors failed to identify systematic biases, and consequent flawed conclusions in TL. Reviews of TL were made public, and one reviewer expressed the hope of seeing TL “…published and used by scientist (*sic*) to promote better policies.” We cannot know whether this reviewer was willing to overlook flaws in TL because of a desire to see it influence policy, but the fact is that the reviewer evidently failed to provide a critical review of the methodology in TL. That such a flawed paper slipped through the review process is problematic, because it will now be cited (and already has been cited in court) as an authoritative source to promote policies that are not supported by more careful science. This underscores our second point. When a paper is published purporting to reach conclusions at odds with most other published research on the topic, it is prudent to avoid making hasty policy decisions based on the conclusions of that paper. The conclusions may be wrong, as they were in the case of TL, and decisions made on the basis of such papers cannot be said to have been made using the best available science.

## Supporting information

S1 File (PDF)
