# Supplementary material for: Uncertainty and precaution in hunting wolves twice in a year: Reanalysis of Treves and Louchouarn
Source: PLoS One. 2024 Jun 12;19(6):e0301487. doi: 10.1371/journal.pone.0301487 (PMC11168653; doi:10.1371/journal.pone.0301487)
Supplement: S1 File — (PDF) [file pone.0301487.s001.pdf]

Uncertainty and precaution in hunting wolves twice in a year: flawed methodology and incorrect conclusions in Treves and Louchouarn

Glenn E. Stauffer, Erik R. Olson, Jerrold L. Belant, Jennifer L. Stenglein, Jennifer L. Price Tack,
Timothy R. van Deelen, David M. MacFarland, Nathan M. Roberts

Supplement S1: R code to replicate Treves and Louchuoarn 2022 and to provide a re-analysis using alternative parameterization

```
####################################################################################
#  Code to replicate Treves and Louchuoarn 2022 (herafter TL), as much as possible
#  and to provide a re-analysis using alternative parameterization
#
# Stauffer et al. 2023
####################################################################################
# packages
library(HDInterval)
library(modeest)
library(dplyr)
library(captioner)
library(xtable)
library(rmarkdown)
library(kableExtra)

#-----------------------------------------------------------------------------------
#-----------------------------------------------------------------------------------
# function for prediction (over range of harvest scenarios)
#-----------------------------------------------------------------------------------
#-----------------------------------------------------------------------------------

f <- function(nsim){
# set random number seed
set.seed(1234)
#------------------------------------------------------
# TL parameterization (as closely as possible)
#------------------------------------------------------
# tribal population to deduct
tribal <- 42

# Feb 2021 harvest
H_Feb2021 <- 218

# initial population size
N2021a <- runif(nsim, 695,751) # from Treves et al. (2021)
N2021a2 <- 1075+(sample(seq(-496,756,1),size=nsim, replace=TRUE)+sample(seq(-
496,756,1),size= nsim,replace=TRUE))/2 - H_Feb2021

# mortality rate (roughly equivalent to D <- runif(nsim, 695,751))
D <- sample(38:56,nsim,replace=TRUE)/100

# number of breeding packs
B <- sample(74:167,nsim,replace=TRUE)

# proportion of packs with pups (roughly a truncated normal with mean 0.68)
PPN <- (((sample(1:17,nsim,replace=TRUE)+sample(1:17,nsim,replace=TRUE))-2)/100)+0.52
```

```
# litter size (this tends to have a mean somewhat lower than specified in the text of
TL)
# Note that adding 0.3 here is completely superflous - it makes no difference
# perhaps TL meant to add 0.5 instead?
L <-floor(((sample(3:6,replace=TRUE)+sample(3:6,nsim,replace=TRUE))/2)+0.3)

# pup survival (right-skewed and truncated at zero, mean 0.21)
S <- ((sample(0:16,nsim,replace=TRUE)+sample(0:16,nsim,replace=TRUE))+
rbinom(nsim,1,0.25)*sample(3:40,nsim,replace=TRUE))/100

# number of pups surviving to fall
r <- PPN*L*S
R <- B*r

# Fall 2021 harvest
H <- sample(0:300,nsim,replace=TRUE)+sample(0:300,nsim,replace=TRUE)

# Non-harvest deaths
M <- D*(N2021a+R/2)
M2 <- D*(N2021a2+R/2)

N2022a <- N2021a+R/2-M-H - tribal # dividing R by 2 here is incorrect
N2022a2 <- N2021a2+R/2-M2-H - tribal # dividing R by 2 here is incorrect

#---------------------------------------------------------
# simulation using alternative numbers (Stauffer et al)
# still considering strictly additive (actually, slightly superadditive) mortality
#---------------------------------------------------------
# tribal population to deduct
tribal <- tribal

# Feb 2021 harvest
H_Feb2021 <- H_Feb2021

# initial population size
N2021b <- runif(nsim, 695,751) # same as TL
N2021b2 <- round(rnorm(nsim,1126,110),0) - H_Feb2021

# mortality rate from Stenglein et al. (2015,2018)
D2 <- rnorm(nsim,0.25,0.019)

# number of breeding packs (total off-rez packs - 51 presumably harvest reproductive
# females). The 51 is almost certainly an overestimate
B2 <- 245-51

# proportion of packs with pups (statistical distribution to approximate TL)
PPN2 <- rbeta(nsim,34.51,16.24)

# litter size (statistical distribution to approximate TL, or actually, Thiel et al.)
# This is a mean, so it does not need to be an integer
# and what is specified here is probably excessively imprecise for a mean
L2 <-rbeta(nsim, 3,2)*(6-3)+3

# pup survival (statistical dist to approximate Table 6.3 of Wydeven et al. (2009),
scaled
# to 7 months)
S2 <- rbeta(nsim,24,25.6) # mean 0.484

# number of pups surviving to fall
r2 <- PPN2*L2*S2
R2 <- B2*r2
```

```
# Fall 2021 harvest
H <- H

# non-harvest deaths
Mb <- N2021b*D2+(1-(1-D2)**(5/12))*(R2-H)
Mb2 <- N2021b2*D2+(1-(1-D2)**(5/12))*(R2-H)

N2022b <- N2021b+R2-Mb-H - tribal
N2022b2 <- N2021b2+R2-Mb2-H - tribal

return(list(N2022a,N2022a2,N2022b,N2022b2,H))
}
# output to generate Figure
out1 <- f(3600)

#-----------------------------------------------------------------------------------------
#-----------------------------------------------------------------------------------------
# function to predict at specific harvest level
#-----------------------------------------------------------------------------------------
#-----------------------------------------------------------------------------------------
f2 <- function(nsim, H){
# set random number seed
set.seed(1234)
#------------------------------------------------------------
# TL parameterization (as closely as possible)
#------------------------------------------------------------
# tribal population to deduct
tribal <- 42

# Feb 2021 harvest
H_Feb2021 <- 218

# initial population size
N2021a <- runif(nsim, 695,751) # from Treves et al. (2021)
N2021a2 <- 1075+(sample(seq(-496,756,1),size=nsim, replace=TRUE)+sample(seq(-
496,756,1),size= nsim,replace=TRUE))/2 - H_Feb2021

# mortality rate (roughly equivalent to D <- runif(nsim, 695,751))
D <- sample(38:56,nsim,replace=TRUE)/100

# number of breeding packs
B <- sample(74:167,nsim,replace=TRUE)

# proportion of packs with pups (roughly a truncated normal with mean 0.68)
PPN <- (((sample(1:17,nsim,replace=TRUE)+sample(1:17,nsim,replace=TRUE))-2)/100)+0.52

# litter size (this tends to have a mean somewhat lower than specified in the text of
TL)
# Note that adding 0.3 here is completely superflous - it makes no difference
# perhaps TL meant to add 0.5 instead?
L <-floor(((sample(3:6,replace=TRUE)+sample(3:6,nsim,replace=TRUE))/2)+0.3)

# pup survival (right-skewed and truncated at zero, mean 0.21)
S <- ((sample(0:16,nsim,replace=TRUE)+sample(0:16,nsim,replace=TRUE))+
rbinom(nsim,1,0.25)*sample(3:40,nsim,replace=TRUE))/100

# number of pups surviving to fall
r <- PPN*L*S
R <- B*r
```

```
# Non-harvest deaths
M <- D*(N2021a+R/2)
M2 <- D*(N2021a2+R/2)

N2022a <- N2021a+R/2-M-H - tribal # dividing R by 2 here is incorrect
N2022a2 <- N2021a2+R/2-M2-H - tribal # dividing R by 2 here is incorrect

#---------------------------------------------------------
# simulation using alternative numbers (Stauffer et al)
# still considering strictly additive (actually, slightly superadditive) mortality
#---------------------------------------------------------
# tribal population to deduct
tribal <- tribal

# Feb 2021 harvest
H_Feb2021 <- H_Feb2021

# initial population size
N2021b <- runif(nsim, 695,751) # same as TL
N2021b2 <- round(rnorm(nsim,1126,110),0) - H_Feb2021

# mortality rate from Stenglein et al. (2015,2018)
D2 <- rnorm(nsim,0.25,0.019)

# number of breeding packs (total off-rez packs - 51 presumably harvest reproductive
# females). The 51 is almost certainly an overestimate
B2 <- 245-51

# proportion of packs with pups (statistical distribution to approximate TL)
PPN2 <- rbeta(nsim,34.51,16.24)

# litter size (statistical distribution to approximate TL, or actually, Thiel et al.)
# This is a mean, so it does not need to be an integer
# and what is specified here is probably excessively imprecise for a mean
L2 <-rbeta(nsim, 3,2)*(6-3)+3

# pup survival (statistical dist to approximate Table 6.3 of Wydeven et al. (2009),
scaled
# to 7 months)
S2 <- rbeta(nsim,24,25.6) # mean 0.484

# number of pups surviving to fall
r2 <- PPN2*L2*S2
R2 <- B2*r2

# non-harvest deaths
Mb <- N2021b*D2+(1-(1-D2)**(5/12))*(R2-H)
Mb2 <- N2021b2*D2+(1-(1-D2)**(5/12))*(R2-H)

N2022b <- N2021b+R2-Mb-H - tribal
N2022b2 <- N2021b2+R2-Mb2-H - tribal

goal <- c(length(which(N2022a <350))/nsim,
          length(which(N2022a2<350))/nsim,
          length(which(N2022b <350))/nsim,
          length(which(N2022b2<350))/nsim)
threat <- c(length(which(N2022a <250))/nsim,
          length(which(N2022a2<250))/nsim,
          length(which(N2022b <250))/nsim,
          length(which(N2022b2<250))/nsim)
extr <- c(length(which(N2022a <2))/nsim,
```

```
          length(which(N2022a2<2))/nsim,
          length(which(N2022b <2))/nsim,
          length(which(N2022b2<2))/nsim)

out <- list(rbind(goal,threat,extr),N2022a,N2022a2,N2022b,N2022b2)
return(out)
}

# hx represent the proportion of simulations (with harvest = x) where the predicted
population was below each of the three population thresholds defined in TL, for 4
situations (initial population from the "count method" and the occupancy model, using
TL parameterization for prediction, or using paramaterization from our re-analysis).
nsim <- 1000000
h0 <-   f2(nsim,0)  [[1]]
h130 <- f2(nsim,130)[[1]]
h300 <- f2(nsim,300)[[1]]
h500 <- f2(nsim,500)[[1]]
h600 <- f2(nsim,600)[[1]]

#!# CHECK - "probability of extirpation was zero for all harvest scenarios"
round(rbind(h0[3,3:4],h130[3,3:4],h300[3,3:4],h500[3,3:4],h600[3,3:4]),2)

#!# CHECK - "Only in scenarios with simulated harvest of 300 or more 300 wolves did
probability of crossing even the first population threshold of 350 exceed zero"
round(rbind(h0[1,3:4],h130[1,3:4],h300[1,3:4],h500[1,3:4],h600[1,3:4]),2)

# For the simulation using the scaled occupancy model for initial population size,
for a zero harvest scenario, calculate predicted ending population size, assuming TL
parameterization or corrected parameterization. Because realized harvest was zero,
these predictions are directly comparable to the spring 2022 scaled occupancy
estimate of 972 (95% CrI 812 - 1193).
tl <-   f2(nsim,0)  [[3]]
gs <-   f2(nsim,0)  [[5]]
TL2022summary <- c(mean(tl),hdi(tl))
GS2022summary <- c(mean(gs),hdi(gs))
write.table(cbind(TL2022summary,GS2022summary),file="PopSizePred.txt",sep=" ",
row.names = FALSE)

#!# CHECK - Compare TL prediction to empirical estimate
round(TL2022summary)
TL2022summary[1]/972

#!# CHECK - Compare corrected prediction to empirical estimate
round(GS2022summary)
GS2022summary[1]/972

#!# CHECK - For harvests of 0, 130, and 300, TL predicted mean population sizes of
361, 231, and 66, respectively, for the "over-winter counts", with SD = 44 - 45 in
all cases; similarly, our duplication of TL predicted population sizes of 360, 230,
and 60, respectively.
sim.h0 <-   f2(nsim,0)
sim.h130 <- f2(nsim,130)
sim.h300 <- f2(nsim,300)

c(round(mean(sim.h0[[2]])),round(sd(sim.h0[[2]])))
c(round(mean(sim.h130[[2]])),round(sd(sim.h130[[2]])))
c(round(mean(sim.h300[[2]])),round(sd(sim.h300[[2]])))

#!# P(pop<350|no harvest)
length(which(sim.h0[[2]]<350))/nsim
```

```
#--------------------------------------------------------------------------------------
----------
# Figure
# Simulations results predicting wolf population size in April 2022, assuming an
uncertain fall harvest of 0--600. The three horizontal lines represent population
thresholds of 350 (1999 population goal), 250 (relisting threshold), and 2
(extirpation) considered in TL.
#--------------------------------------------------------------------------------------
----------
{
x11(width=6.5,height=6.5)
H <- out1[[5]]
# min count
par(mfrow=c(2,2),mar=c(1,1,0.5,0), oma=c(2.5,2.5,1,1))
plot(H,out1[[1]],ylim=c(-300,1300),main = "",
xlab="",ylab="",xaxt="n",pch=20,cex=0.25); abline(h=2,col="red");
abline(h=250,col="blue"); abline(h=350,col="gray")
axis(side=1,at=seq(0,600,100),labels=FALSE)
text(1,1225,"TL, count estimate",adj=0)

#mtext(side=3,line=1,outer=FALSE,text="TL")

plot(H,out1[[3]],main = "", ylim=c(-
300,1300),xlab="",ylab="",yaxt="n",xaxt="n",pch=20,cex=0.25); abline(h=2,col="red");
abline(h=250,col="blue"); abline(h=350,col="gray",ylab="",xlab="")
axis(side=1,at=seq(0,600,100),labels=FALSE)
text(1,1225,"Re-analysis, count estimate",adj=0)

#mtext(side=3,line=1,outer=FALSE,text="Corrected")

# occupancy model estimates
plot(H,out1[[2]],ylim=c(-300,1300),main = "",xlab="",ylab="",pch=20,cex=0.25);
abline(h=2,col="red"); abline(h=250,col="blue"); abline(h=350,col="gray")
text(1,1225,"TL, scaled occupancy estimate",adj=0)

plot(H,out1[[4]],ylim=c(-300,1300),main =
"",xlab="",ylab="",yaxt="n",pch=20,cex=0.25); abline(h=2,col="red");
abline(h=250,col="blue"); abline(h=350,col="gray",ylab="",xlab="")
text(1,1225,"Re-analysis, scaled occupancy estimate",adj=0)

mtext(side=1,line=1.25,outer=TRUE,text="Harvest, fall 2021")
mtext(side=2,line=1.5,outer=TRUE,text="Population size, 2022")

#-------
# pdf
#-------
pdf(file = "predictions.pdf",height=6.5,width=6.5)
# min count
par(mfrow=c(2,2),mar=c(1,1,0.5,0), oma=c(2.5,2.5,2.5,1))
plot(H,out1[[1]],ylim=c(-300,1300),main = "",
xlab="",ylab="",xaxt="n",pch=20,cex=0.25); abline(h=2,col="red");
abline(h=250,col="blue"); abline(h=350,col="gray")
axis(side=1,at=seq(0,600,100),labels=FALSE)
text(1,1225,"TL, count estimate",adj=0)
mtext(side=3,line=1,outer=FALSE,text="TL")
plot(H,out1[[3]],main = "", ylim=c(-
300,1300),xlab="",ylab="",yaxt="n",xaxt="n",pch=20,cex=0.25); abline(h=2,col="red");
abline(h=250,col="blue"); abline(h=350,col="gray",ylab="",xlab="")
axis(side=1,at=seq(0,600,100),labels=FALSE)
text(1,1225,"Re-analysis, count estimate",adj=0)
```

```
mtext(side=3,line=1,outer=FALSE,text="Corrected")
# occupancy model estimates
plot(H,out1[[2]],ylim=c(-300,1300),main = "",xlab="",ylab="",pch=20,cex=0.25);
abline(h=2,col="red"); abline(h=250,col="blue"); abline(h=350,col="gray")
text(1,1225,"TL, scaled occupancy estimate",adj=0)
plot(H,out1[[4]],ylim=c(-300,1300),main =
"",xlab="",ylab="",yaxt="n",pch=20,cex=0.25); abline(h=2,col="red");
abline(h=250,col="blue"); abline(h=350,col="gray",ylab="",xlab="")
text(1,1225,"Re-analysis, scaled occupancy estimate",adj=0)
mtext(side=1,line=1.25,outer=TRUE,text="Harvest, fall 2021")
mtext(side=2,line=1.5,outer=TRUE,text="Population size, 2022")
dev.off()

#-------
# eps
#-------
setEPS()
postscript(file = "predictions.eps",height=6.5,width=6.5)
# min count
par(mfrow=c(2,2),mar=c(1,1,0.5,0), oma=c(2.5,2.5,2.5,1))
plot(H,out1[[1]],ylim=c(-300,1300),main = "",
xlab="",ylab="",xaxt="n",pch=20,cex=0.25); abline(h=2,col="red");
abline(h=250,col="blue"); abline(h=350,col="gray")
axis(side=1,at=seq(0,600,100),labels=FALSE)
text(1,1225,"TL, count estimate",adj=0)
mtext(side=3,line=1,outer=FALSE,text="TL")
plot(H,out1[[3]],main = "", ylim=c(-
300,1300),xlab="",ylab="",yaxt="n",xaxt="n",pch=20,cex=0.25); abline(h=2,col="red");
abline(h=250,col="blue"); abline(h=350,col="gray",ylab="",xlab="")
axis(side=1,at=seq(0,600,100),labels=FALSE)
text(1,1225,"Re-analysis, count estimate",adj=0)
mtext(side=3,line=1,outer=FALSE,text="Corrected")
# occupancy model estimates
plot(H,out1[[2]],ylim=c(-300,1300),main = "",xlab="",ylab="",pch=20,cex=0.25);
abline(h=2,col="red"); abline(h=250,col="blue"); abline(h=350,col="gray")
text(1,1225,"TL, scaled occupancy estimate",adj=0)
plot(H,out1[[4]],ylim=c(-300,1300),main =
"",xlab="",ylab="",yaxt="n",pch=20,cex=0.25); abline(h=2,col="red");
abline(h=250,col="blue"); abline(h=350,col="gray",ylab="",xlab="")
text(1,1225,"Re-analysis, scaled occupancy estimate",adj=0)
mtext(side=1,line=1.25,outer=TRUE,text="Harvest, fall 2021")
mtext(side=2,line=1.5,outer=TRUE,text="Population size, 2022")
dev.off()

}


#--------------------------------------------------------------------------------
----------
# Table
# Comparison of model parameterization from Treves and Louchouarn (2021) with values
used in this paper for reanalysis."
#--------------------------------------------------------------------------------
----------
{
vars <-
c("$N_{t}$","$N_{t}$",'\\textit{D}',"$B_{t}$",'\\textit{PPN}','\\textit{L}','\\textit
{S}',"$R_{t}$",'\\textit{H}',"$M_{t}$","$N_{t+1}$")
Description <- stringr::str_wrap(c("Initial N (min count)","Initial N (occupancy
model)","Annual mortality rate","Packs in population","Proportion of packs
```

```
reproducing","Litter size","Pup survival to Nov","Total pups surviving to
Nov","Harvest","Non-harvest deaths","Ending population"))
TL <- stringr::str_wrap(c('\\textit{U}(695,751)',
        "$1075+(dU(-496,756)+dU(-496,756))/2-218$",
        '\\textit{U}(0.38,0.56)',
        '\\textit{dU}(74,167)',
        "$\\lbrace (dU(1,17)+dU(1,17))-2 \\rbrace/100+0.52$",
        "$\\lbrace TRUNC(dU(3,6)+dU(3,6) \\rbrace/2+0.3)^a$",
        "$\\lbrace (dU(0,16)+dU(0,16)+Bern(0.25) \\times dU(3,40) \\rbrace/100$",
        "$B_t \\times \\textit{PPN} \\times \\textit{L} \\times \\textit{S}$",
        "$dU(0,300)+dU(0,300)$",
        "$D \\times (N_t + R_t/2)$",
        "$N_t +R_t/2-M_t-H-tribal$"))
US <- stringr::str_wrap(c('Same',
        "$N(1126,110)$",
        '\\textit{N}(0.25,0.019)',
        '245 - 51 = 194',
        "$Be(34.51,16.24)$",
        "$Be(3,2) \\times (6-3) + 3$",
        '$Be(24,25.6)$',
        'Same',
        'Same',
        "${M_{t1}+M_{t2}}^b$",
        "$N_t +R_t-M_t-H-tribal$"))
Rationale <- stringr::str_wrap(c("For illustration, we used the values from TL,
although these values do not DNR represent over-winter counts.","TL specification has
fat tails, resulting in bias toward extreme values - our specification closely
approximates the posterior distribution for the DNR 2021 population estimate.",
                      "Stenglein et al. (2015, 2018) provided more plausible and
defensible mortality estimates.",
                      'TL twice accounted for non-breeding packs, and $B_t$ = 74
represents improper and misleading extrapolation from a very small sample.',
                      "Standard statistical distribution (mean = 0.68) to approximate
TL",
                      "Standard statistical distribution (mean = 4.8) to approximate
\\cite{thielDisjunctGrayWolf2009}",
                      "Standard statistical distribution (mean = 0.484) that
approximates the histogram of annual survival, scaled to 7 months, from Table 6.3 of
\\cite{wydevenHistoryPopulationGrowth2009}.",
                      "We accept the equation from TL.",
                      "We used the harvest scenarios from TL.",
                      "TL improperly counted some deaths twice.",
                      "TL improperly doubled pup mortality (i.e., $R_t/2$)."))
tab <- cbind(vars,Description,TL,US,Rationale)
colnames(tab) <- c("Parameter","Description","TL","This paper","Rationale")
cap1 <- "{\\bf Comparison of model parameterization from Treves and
Louchouarn~\\cite{trevesUncertaintyPrecautionHunting2022} with values used in this
paper for reanalysis. In parameter notation, U represents a uniform distribution, dU
represents a discrete uniform distribution (integer values between specified bounds),
N represents a normal distribution, Be represents a beta distribution, and Bern
represents a Bernoulli distribution.}"
row.names(tab) <- NULL

tab2 <-
xtable(x=tab,caption=cap1,label="tab:Parmtab",align=c("l","p{1.5cm}","p{3.5cm}","p{3c
m}","p{3cm}","p{8cm}"))

add = list(pos=list(11,11), command=
             c("\\hline\\multicolumn{5}{l}{\\textsuperscript{a} Note that the
addition of 0.3 here does not affect the truncated value.}\\","
```

```
\\multicolumn{5}{l}{\\textsuperscript{b}$M_{t1}+M_{t2} = N_t \\times D + [1-(1-
D)^{5/12}] \\times (R_t -H)$} \\\\"))

print(tab2,
        table.placement="!ht",
        caption.placement="top",
        include.rownames = FALSE,
        sanitize.text.function=function(x){x},
        add.to.row=add,
        hline.after=c(-1,0))

}


#-----------------------------------------------------------------------------------------------
----------
# Table
# Threshold crossing probabilities
#-----------------------------------------------------------------------------------------------
----------
{
Harvest <- rep("",times=3*5)
Threshold <- rep(c("Management","Relisting","Extirpation"),times=5)
probs <- rbind(h0,h130,h300,h500,h600)
dff <- cbind(Harvest,Threshold,formatC(round(probs,3),digits=3,format="f"))
dff[3,2] <- "Extirpation\u200c" # because kableExtra inserts an unwanted space when
rows are identical
#dff[3,6] <- "0.000\\vphantom{1}" # This seems to not work anymore

colnames(dff) <- stringr::str_wrap(c("Harvest", "Threshold\\textsuperscript{a}","TL -
Count","TL - Occ","Current - Count","Current - Occ"))
cap <- "{\\bf Probability of crossing 3 population thresholds defined in Treves and
Louchouarn (TL)~\\cite{trevesUncertaintyPrecautionHunting2022} for various fall 2021
harvest scenarios when starting population is estimated using the traditional (Count)
method or occupancy model method (Occ; Stauffer et
al.~\\cite{staufferScalingOccupancyEstimates2021}), and using parameterization from
TL or corrected parameterization from this paper (Current). Note that the minimums
count used in TL is not directly comparable to counts generated by the Wisconsin
Department of Natural Resources prior to 2021, but rather represents deterministic
calculations resulting from assumptions about assumed hypothetical population growth
rates \\cite{trevesQuantifyingEffectsDelisting2021}. For this comparison, we used the
values for pup survival provided in the original TL manuscript, and not the values
listed in the correction issued in a comment on TL, because the comment did not
explain clearly what distribution was used in the correction. TL stated that their
conclusions did not change, but we note that probabilities of crossing below
threshholds were slightly reduced after the correction.}"
row.names(dff) <- NULL

tab3 <-
xtable(x=dff,caption=cap,label="tab:Probtab",align=c("l","p{1.75cm}","p{3cm}",rep("p{
1.75cm}",4)))

add = list(pos=list(0,3,6,9,12,15), command= c("\\hline \\multicolumn{6}{l}{Harvest =
0}\\\\",

      paste0("\\multicolumn{6}{l}{Harvest = ",c(130,300,500,600),"}\\\\"),
                                                                         "\\hline
\\multicolumn{6}{l}{\\textsuperscript{a}Thresholds were: Management, 350; Relisting,
250; Extirpation, \\textless{2}}\\\\"))

print(tab3,
```

```
        table.placement="!ht",
        caption.placement="top",
            include.rownames = FALSE,
        sanitize.text.function=function(x){x},
        add.to.row=add,
        hline.after=c(-1))

}
```
